# Management of Complications Following Botulinum Toxin Facial Injections: A Narrative Review

**DOI:** 10.7759/cureus.100675

**Published:** 2026-01-03

**Authors:** Sara Freixo, Alexandre Camões-Barbosa

**Affiliations:** 1 Department of Physical Medicine and Rehabilitation, Hospital de Braga, Braga, PRT; 2 Clinical Neurophysiology Unit, Centro Hospitalar Universitário de Lisboa Central, Lisbon, PRT

**Keywords:** adverse effects, blepharoptosis, botulinum toxin, botulinum toxin injection, diplopia, drug-related side effects, treatment options

## Abstract

Botulinum toxin (BoNT) is widely used in the management of neurological disorders and in aesthetic medicine. Although generally safe, facial injections may be associated with complications with relevant functional or aesthetic impact. This narrative review aimed to summarize reported facial complications following BoNT injections and to describe available management strategies.

A PubMed search up to November 2025 identified 239 articles; after screening and full-text review, 20 studies met the inclusion criteria. Data were synthesized narratively due to the heterogeneity of study designs.

Upper eyelid ptosis was the most frequently reported clinically significant complication and was mainly managed with topical alpha-adrenergic agonists. Diplopia was rare but functionally disabling and was treated conservatively with occlusion or prisms or with targeted extraocular BoNT injection in selected cases. Ocular surface changes, facial asymmetry, perioral dysfunction, local reactions, and headache were generally mild and self-limited. Systemic adverse events were uncommon but occasionally required hospital evaluation. Overall, management strategies were predominantly conservative and supported by low-level evidence.

Facial BoNT injections are generally safe, but clinically relevant complications can occur. Management is largely conservative, apraclonidine 0.5% is most commonly used for toxin-induced ptosis, and oxymetazoline 0.1% (FDA-approved for acquired blepharoptosis) is an additional option; other events are treated symptomatically. Overall, evidence is limited, supporting the need for prospective studies and standardized management pathways.

## Introduction and background

Botulinum toxin (BoNT) is widely used in the management of neurological disorders, particularly within physical and rehabilitation medicine and neurology, where it is a key treatment option for conditions such as blepharospasm, hemifacial spasm, facial dystonia, and headache disorders [1-3].

The field of clinical applications is increasing with reports of efficacy in other disorders, such as temporomandibular joint disorder, neuropathic pruritus, spasticity-associated pain, thoracic outlet syndrome, and many more [4-9]. In addition to these therapeutic indications, BoNT is also extensively employed in aesthetic medicine, particularly for facial rejuvenation, resulting in a broad spectrum of facial injection practices across medical specialties [10,11].

Clinical series and consensus reviews report that BoNT is effective and generally well tolerated across these indications, with most adverse events being transient and mild to moderate [10,12-14], even in older patients [15].

A recent systematic review and meta-analysis of BoNT face injections found an overall complication rate of approximately 12-16%, with headache, local skin reactions, and neuromuscular symptoms as the most frequent events and very few severe treatment-related complications [10]. 

Therapeutic series in blepharospasm and hemifacial spasm similarly describe low but clinically relevant rates of complications that are usually dose-related and reversible [1,2].

Despite this reassuring profile, BoNT face injections can occasionally be associated with more serious or functionally significant complications, including eyelid or brow ptosis, facial paresis, diplopia, ocular surface alterations, perioral dysfunction, local reactions, headache, and systemic adverse events [16-18].

For physiatrists, neurologists, and other clinicians who routinely inject BoNT in the face, these complications may have meaningful functional and psychological consequences.

Periocular problems such as ptosis or diplopia can impair visual tasks, including reading, driving, and balance-dependent activities [19,20]. Perioral weakness may interfere with speech, articulation, and oral competence, particularly in patients with pre-existing neurological impairment [11,18]. Even complications that are primarily cosmetic, such as eyebrow deformities or facial asymmetry, may cause distress and reduced patient satisfaction [12,21]. Although usually self-limited, these events, even when uncommon, may exert a disproportionate impact on daily functioning and overall quality of life [19].

Several reviews and consensus documents have addressed the prevention of complications related to BoNT injections, emphasizing patient selection, detailed knowledge of facial anatomy, appropriate dosing, and meticulous injection technique [12,22,23]. In contrast, comparatively little attention has been given to the management of complications once they occur, and structured, evidence-based guidance for their treatment remains limited.

The aim of this narrative review is to summarize the available evidence on facial complications related to BoNT and to describe reported management strategies and outcomes.

## Review

Methodology

A literature search was conducted in PubMed in November 2025 using a predefined search strategy (Botulinum Toxins, Type A”[MeSH] OR botulinum) AND (complications AND (management OR treatment)) AND (“Face”[MeSH] OR facial) and limited to human studies and English-language articles.

**Figure 1:**
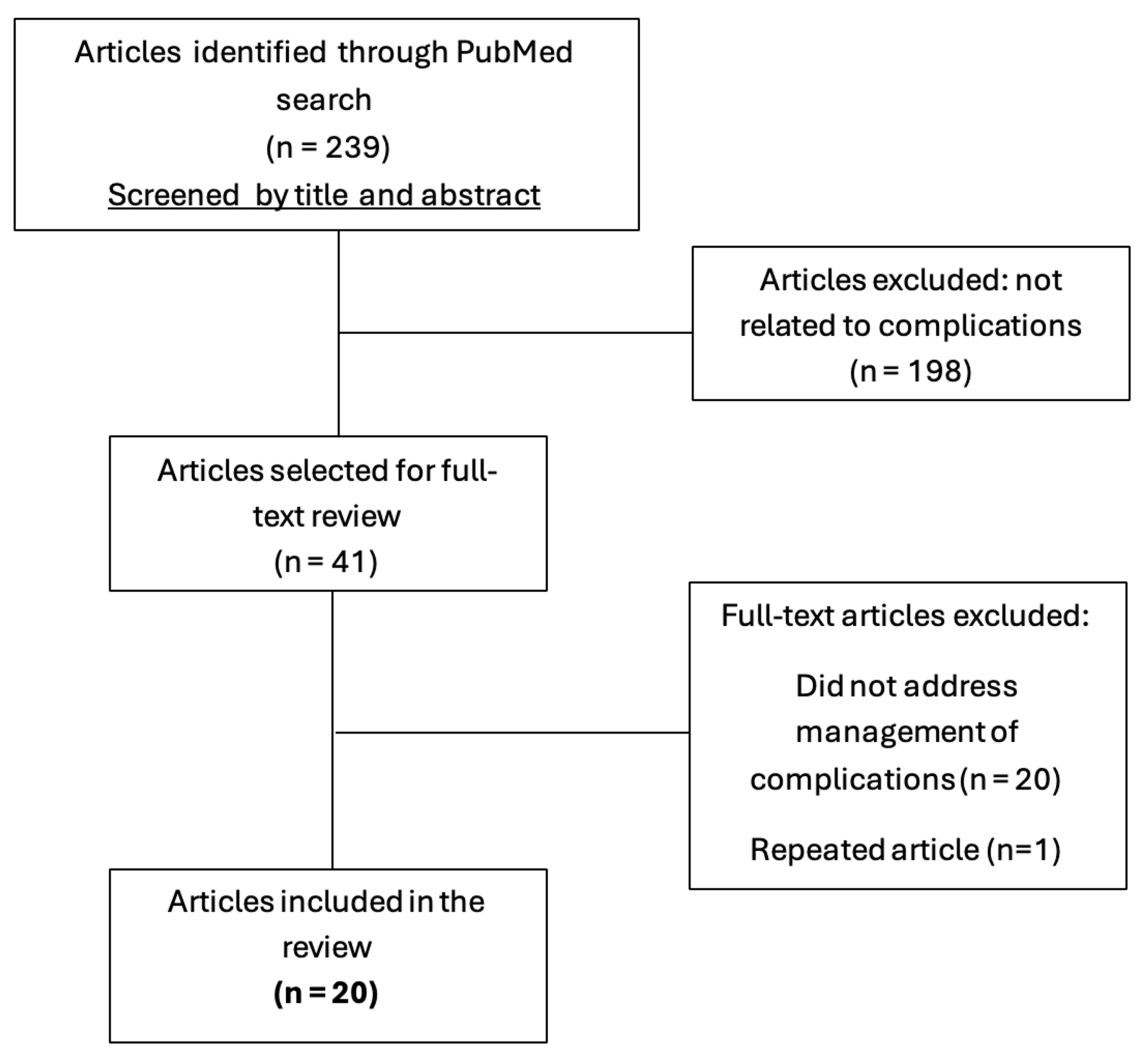
Flow diagram of the literature search and study selection process

The search retrieved 239 articles. The study selection process is summarized in Figure 1. Titles and abstracts were screened to identify studies reporting complications related to facial BoNT injections. Articles that clearly did not address complications or dealt exclusively with efficacy, technical aspects, or non-facial indications were excluded, leaving 41 articles for full-text review.

Inclusion criteria were as follows: use of BoNT injections in the face; description of at least one treatment-related complication; and some description of management or clinical course of these complications (therapeutic interventions, conservative management, or outcomes). Both primary studies and narrative reviews were eligible for inclusion. Articles were excluded if they did not relate to facial injections or if they mentioned complications without providing any information on their management. This review focused exclusively on complications related to BoNT type A.

After full-text assessment, 20 articles met the inclusion criteria and were included in the qualitative synthesis.

For each included article, data were extracted on study type, complications reported, incidence or frequency when available, management strategies, and clinical outcomes. Owing to the heterogeneity of designs, ranging from case reports and narrative reviews to pharmacovigilance database analyses, a descriptive narrative synthesis was performed.

Complications were grouped as ptosis, diplopia, ectropion, ocular surface changes, facial asymmetry, perioral dysfunction, local injection-site reactions, and systemic adverse events. Complications were classified according to the definitions used in the original reports. These methodological choices are acknowledged as limitations of this narrative review. 

Due to feasibility and practical considerations, the search was restricted to this single database. PubMed was selected because it is the most widely used biomedical database and was considered an appropriate source to identify clinically relevant literature across the specialties involved in facial BoNT practice. This approach may have resulted in the omission of relevant studies indexed elsewhere and therefore represents a limitation of this review. Study screening and selection were performed by a single reviewer, without a formal inter-rater reliability assessment. This approach may introduce selection bias and is acknowledged as a limitation of this narrative review. Protocol registration was not performed, which is acknowledged as a limitation of this narrative review.

When overlapping information was identified across narrative reviews and primary reports, original source articles were prioritized, and findings were synthesized descriptively.

Results

Study Characteristics

A total of 20 articles met the inclusion criteria and were included in the qualitative synthesis. Most were narrative review papers from dermatology, plastic surgery, maxillofacial surgery, and ophthalmology, frequently combining technique description with discussion of complications and their prevention. Six included papers reported clinical cases with facial BoNT complications. One systematic review specifically examined ophthalmic adverse events after facial BoNT injections, and one pharmacovigilance study analyzed spontaneous reports of cosmetic BoNT adverse events from the Food and Drug Administration (FDA) database. Overall, the available evidence was dominated by expert opinion and retrospective data rather than prospective comparative trials, which limited the direct comparison or ranking of management strategies across complication types.

Upper Eyelid Ptosis

Upper eyelid ptosis was the most frequently described clinically significant complication. Narrative reviews and clinical series reported rates ranging from about 1-14% after glabellar injections, decreasing to <1% in series from experienced injectors, highlighting heterogeneity likely related to injector experience, technique, dose, as well as differences in patient factors and indications [11,16,24,25]. In the pharmacovigilance study of 10577 cosmetic BoNT reports, eyelid and brow ptosis together accounted for 6.1% of all adverse events [17].

BoNT-A-induced upper eyelid ptosis is believed to result from diffusion of toxin into the orbit, where it weakens the levator palpebrae superioris. Several mechanisms have been proposed, including penetration or seepage through the orbital septum, migration along areas of septal dehiscence, and spread via the supraorbital or supratrochlear foramina. This risk increases when injections are placed close to the supraorbital margin, particularly along the mid-pupillary line, or when higher volumes or deeper injections are used and may be further accentuated in older patients with levator atrophy or septal attenuation [11,16,18,26].

Clinically, toxin-induced upper eyelid ptosis typically appears within the first week after injection, up to about 7-10 days, and most cases resolve within 2-4 weeks, although some reports describe a more prolonged course, without specifying a maximum duration [11,16,20].

Across the included articles, first-line management consisted of topical alpha-adrenergic agonist eye drops, most commonly apraclonidine 0.5%, typically administered as one to two drops three to four times daily until the resolution of ptosis, with some authors advising reassessment after 20-30 minutes for possible additional instillation, reflecting expert practice rather than standardized protocols [24,26-28]. One included review also noted that oxymetazoline 0.1% has been approved by the FDA for the treatment of acquired blepharoptosis, citing two randomized phase III trials in which once-daily instillation produced significant improvements in eyelid elevation and superior visual field [28]. Alternative agents such as phenylephrine 2.5%, naphazoline, or brimonidine were mentioned as options, depending on availability and ocular comorbidities [16,18,29]. Topical alpha-adrenergic agonists improve toxin-induced eyelid ptosis by stimulating Müller's muscle, providing 1-2 mm of compensatory eyelid elevation while levator function recovers [18,27]. Use of alpha-adrenergic agents should take into account patient-specific contraindications and comorbidities. Mild ptosis may be managed expectantly, whereas more pronounced or visually significant cases sometimes receive adjunctive measures such as lymphatic drainage, low-voltage electrical stimulation, or radiofrequency [19,26]. However, although these modalities are mentioned in some reports, the articles do not provide supporting evidence, methodological detail, or citations to substantiate their efficacy and therefore cannot be recommended as standard management. One narrative review described a multimodal approach combining apraclonidine 0.5% with physical modalities and, in selected expert-level contexts, pretarsal BoNT injection to rebalance orbicularis oculi activity; however, the authors did not provide any details regarding injection sites, technique, or dosing [26]. Overall, all reports described full or near-full resolution over a few weeks to months.

Diplopia

Diplopia and ocular motility disturbances were described as rare complications, generally attributed to unintended toxin spread to extraocular muscles (most commonly the lateral rectus) after periocular injections [16,25,29]. Narrative reviews estimated diplopia rates below 1% when injections were performed by experienced practitioners, although precise incidence data are limited and may be influenced by the under-recognition of transient symptoms and reporting bias [16,25]. The systematic review by Skorochod et al. identified 49 published cases of ophthalmic adverse events after facial BoNT, and diplopia was the most frequent manifestation, usually binocular and associated with difficulties in reading, driving, and maintaining balance [20]. In that review, most patients recovered over weeks, in line with the expected duration of BoNT-A activity.

Some reports described conservative management with temporary occlusion, prism glasses, and observation, with recovery generally occurring within 6-10 weeks [19,20]. Some reviews emphasized the need for ophthalmologic assessment to exclude other causes and to individualize decisions on occlusion, prisms, or therapeutic extraocular chemodenervation [25,29]. Two case reports provided more detailed management strategies: both described lateral rectus paresis after cosmetic periocular BoNT-A, successfully treated with 2.5 U (0.05 ml) of onabotulinumtoxinA into the ipsilateral medial rectus muscle using a transconjunctival approach with topical anaesthesia and Mendonça's forceps, leading to substantial improvement within 2-7 days and complete resolution on follow-up. This approach requires specialist ophthalmologic expertise and may not be generalizable to routine practice [30].

Ectropion

Ectropion was mentioned less frequently than ptosis or diplopia but was highlighted as a potentially vision-threatening event if associated with exposure keratitis. Vartanian and Dayan reported ectropion after lower-lid or lateral canthal BoNT-A injections, linked to the excessive weakening of the pretarsal orbicularis. Management in these reports consisted of intensive lubrication with topical preservative-free artificial tears and ointments, combined with temporary lid taping to prevent exposure keratopathy while awaiting the recovery of orbicularis function [25]. No surgical interventions were reported as necessary in the included literature; however, it is unclear whether this reflects a true lack of clinical need or under-reporting in the available literature.

Ocular Surface Changes (Dry Eye)

Ocular surface alterations, particularly dry eye, were described in case reports and small series following lateral canthal injections. Matarasso reported a patient who developed a unilateral reduction in Schirmer test values and superficial punctate keratopathy after the treatment of lateral canthal rhytides. The patient complained of foreign-body sensation and pain [31]. Management consisted of preservative-free artificial tears and ocular surface protection, with the normalization of Schirmer values and the resolution of keratopathy over four months. Matarasso noted that although BoNT could theoretically affect tear production through diffusion to the lacrimal gland, this mechanism is considered unlikely because the gland is deeply protected within the lacrimal fossa of the orbital roof and is largely shielded by the orbital portion of the frontal bone. Instead, the more plausible explanation for dry eye symptoms relates to the inadvertent chemodenervation of the pretarsal orbicularis oculi. The pretarsal fibers play a critical role in the lacrimal pump, generating the pressure changes that drive tear movement into and through the nasolacrimal system. Paresis of these fibers impairs nasolacrimal outflow and leads to functional tear deficiency [31].

Subsequent BoNT sessions were carried out with a modified technique, avoiding the lateral canthus, without recurrence. Ferreira et al. described a similar presentation of dry eye and discomfort, managed with lubricants and topical anti-inflammatory drops, with symptomatic resolution after approximately 60 days [19]. Narrative reviews from dermatology and ophthalmology confirmed that eye-surface complications appear rare and are mostly reported as isolated cases, but emphasized the risk of corneal damage if missed, recommending the early use of frequent tear substitutes and the adjustment of subsequent injection patterns [16,29].

Facial Asymmetry

Facial asymmetry, including undesired eyebrow shapes such as the "Spock" or "Mephisto" brow, was described as relatively common but seldom quantified, potentially leading to under-recognition despite its impact on patient satisfaction. Reviews attributed these problems to uneven dosing, failure to account for baseline asymmetry, or excessive paralysis of some frontalis segments [16,22,27,28]. Clinical impact was mainly cosmetic, with altered facial expression and patient dissatisfaction [16]. Management consisted of reassurance and observation in mild cases, as asymmetry tends to diminish as toxin effects wear off, or of small corrective BoNT doses into the relatively overactive muscle [27,28].

Perioral Dysfunction (Lip Ptosis, Oral Incompetence, Drooling)

Perioral dysfunction was consistently identified as a high-risk complication area because of its impact on speech and feeding. Narrative reviews described lip ptosis, oral incompetence, and drooling as rare in aesthetic practice but potentially disabling, particularly in patients with pre-existing neurological disease [11,18,22]. Although precise incidence is unknown, the literature consistently notes that misplaced or excessive dosing around the orbicularis oris can lead to noticeable functional impairment [18]. Clinical manifestations included difficulty retaining liquids, slurred speech, and drooling, with negative social and psychological consequences [16].

Across the included literature, management of perioral dysfunction was almost exclusively conservative, which may reflect the absence of established corrective interventions rather than evidence that conservative care is optimal. The authors mainly recommended explanation, reassurance, and expectant recovery while awaiting the resolution of the toxin effect [11,18]. In clinical practice, additional compensatory strategies such as using straws, adjusting head position when drinking, or involving speech therapy may be used to minimize functional impact.

Local Injection-Site Reactions (Pain, Erythema, Oedema, Bruising)

Local injection-site reactions, including pain, erythema, oedema, and ecchymosis, were the most frequently reported adverse events in clinical trials and pharmacovigilance data. Narrative reviews described these events as common but usually mild and short-lived, often under-reported in the literature [22,27,32]. In the pharmacovigilance data, pain, swelling, erythema, and bruising represented 9.3%, 6.4%, 1.8%, and 1.2% of reported cosmetic BoNT adverse events, respectively. Clinical impact was generally limited to transient discomfort and cosmetic concern but could influence patients' willingness to undergo repeated treatments [22,27].

Management was symptomatic in all sources. Recommended measures included ice packs, gentle compression to reduce bruising, elevation of the head for eyelid oedema, and simple analgesics when needed [22,27,32]. Preventive strategies focused on using fine-gauge needles, injecting slowly with small volumes, avoiding unnecessary antithrombotic medication where possible, and applying brief pressure after each injection [10,17].

Headache

Headache was a frequently mentioned adverse event, although a direct causal association with BoNT cannot be clearly established. Available reports suggest that post-injection headache is generally mild to moderate and self-limiting; however, overlap with pre-existing or coincidental headache disorders makes attribution to BoNT exposure challenging and precludes firm conclusions regarding treatment-specific risk [22,27]. In the severe complication series by Ferreira et al., two of eight patients presented with intense bilateral headache lasting 15-45 days after treatment, which required oral analgesics for control but resolved without sequelae [19]. According to the pharmacovigilance analysis, headache constituted 4.3% of the adverse events reported after cosmetic BoNT injections [17].

All sources recommended symptomatic management using nonsteroidal anti-inflammatory drugs or other analgesics and clinical review if the headache was prolonged, atypical, or accompanied by other neurological signs [19,22,27].

Other Events (Fatigue, Flu-Like Symptoms, Weakness, Dyspnoea, Dysphagia, Dizziness, Allergy)

Systemic or generalized adverse events were reported as uncommon but potentially serious. Narrative reviews and pharmacovigilance data described fatigue, flu-like symptoms, malaise, generalized weakness, dyspnoea, dysphagia, dizziness, and allergic reactions, including urticaria and rash, after BoNT injections, often in patients with multiple risk factors or high cumulative doses [12,18,22,33]. In the pharmacovigilance cohort, fatigue, influenza-like illness, muscle weakness, dyspnoea, and dysphagia accounted for 2.8%, 1.4%, 1.2%, 1.3%, and 1.2% of reported events, respectively, while allergy or urticaria represented 3.4% [17]. Notably, among cases with a reported adverse event, 13.5% were classified as serious, such as hospitalization, underscoring that systemic presentations, although uncommon, may carry meaningful clinical risk, particularly in patients with predisposing factors or higher cumulative exposure [17]. The articles provided limited detail on specific treatment protocols for these systemic events. In general, the authors emphasize supportive care and close clinical monitoring, with a low threshold for hospital admission [17,18].

The main characteristics of the included studies are summarized in Table 1.

**Table 1:** Main characteristics of the included studies BoNT: botulinum toxin

Author (year)	Field/main context	Study design	Main focus related to facial BoNT
Başar and Arıcı (2016) [1]	Ophthalmology	Narrative review	Uses of BoNT in ophthalmology and facial aesthetics
Ferreira and Camões-Barbosa (2004) [4]	Aesthetic surgery	Case series	Eight severe complications after facial BoNT
Stephan and Wang (2011) [11]	Facial plastic surgery	Narrative review	Techniques, applications, and complications of BoNT in the treatment of facial and neck muscles
Kroumpouzos et al. (2021) [16]	Cosmetic dermatology	Narrative review	Update on complications of BoNT
Lee et al. (2020) [17]	Maxillofacial surgery	Pharmacovigilance study	Report the most commonly reported adverse events with cosmetic BoNT injections
Klein (2004) [18]	Dermatology	Narrative review	Contraindications and complications of BoNT
Skorochod et al. (2021) [20]	Cosmetic dermatology	Systematic review	Ophthalmic adverse events after facial BoNT injections
Niamtu (2003) [21]	Oral and maxillofacial surgery	Case series	BoNT cosmetic treatments: safety and efficacy assessment
Hassouneh and Newman (2013) [22]	Plastic surgery	Narrative review	Avoiding complications with lasers, fillers, and neurotoxins in facial aesthetics
Nettar and Maas (2012) [23]	Plastic surgery	Narrative review	Complications of facial fillers and neurotoxins
Carucci and Zweibel (2001) [24]	Plastic surgery	Narrative review	Use of BoNT in facial rejuvenation: technique for the prevention of adverse effects
Vartanian and Dayan (2005) [25]	Facial plastic surgery	Narrative review	Complications of BoNT in facial rejuvenation
Jaime Alberto et al. (2025) [26]	Dermatology	Narrative review/case report	Multimodal management of iatrogenic blepharoptosis
Nanda and Bansal (2013) [27]	Aesthetic surgery	Narrative review	Technique and complications in the treatment of upper-face rejuvenation with BoNT and fillers
Johnson and Chen (2023) [28]	Plastic surgery	Narrative review	Cosmetic BoNT injections: management of complications
Nagendran et al. (2022) [29]	Ophthalmology	Narrative review	Complications and adverse effects of periocular treatments
Isaac et al. (2012) [30]	Ophthalmology	Case report	Diplopia after cosmetic BoNT treated with extraocular BoNT
Matarasso (2002) [31]	Dermatology	Case report	Decreased tear production after lateral canthal BoNT
Small (2014) [32]	General medicine/aesthetics	Narrative review	BoNT injection for facial wrinkles: technique and complications
Nittari et al. (2025) [34]	Clinical pharmacology	Narrative review/case report	BoNT-induced blepharoptosis: anatomy, aetiology, and management

Table 2 provides an overview of complications by anatomical/functional category.

**Table 2:** Complications of BoNT facial injections reported in the included studies BoNT: botulinum toxin; FDA: Food and Drug Administration; NSAIDs: nonsteroidal anti-inflammatory drugs

Complication category	Incidence/frequency	Clinical impact	First-line management	Articles reporting
Upper eyelid ptosis	1-14%. ≈1% with experienced injectors	Visual disturbance, cosmetic distress, temporary limitation of daily activities; usually reversible within 6-12 weeks	First-line management is topical apraclonidine 0.5% used several times daily until resolution. Alternative alpha-adrenergic agonists include phenylephrine 2.5%, naphazoline, oxymetazoline, or brimonidine. Mild cases may be managed expectantly, while selected cases may require adjunctive measures such as pretarsal BoNT (expert injector only)	[24,16,32,11,25,18,27,23,22,28,34,21,4,26,17]
Diplopia	Rare; <1% if experienced practitioner	Binocular diplopia, driving and reading difficulty, imbalance; usually resolves over weeks but can be highly disabling	Ocular occlusion, glasses with prismatic lenses, or use of BoNT injection into the antagonist muscle	[25,20,30,17]
Ectropion	Not mentioned	Exposure keratitis, ocular discomfort	Prevent exposure keratitis and corneal damage with topical lubricant drops and lid taping	[25]
Ocular surface changes (dry eye)	Rare, no robust incidence	Foreign-body sensation, pain, superficial keratopathy; risk of corneal damage if unrecognized	Preservative-free artificial tears, anti-inflammatory drops, and ocular surface protection	[31,4]
Facial asymmetry	Relatively common but not consistently quantified	Cosmetic displeasure, altered facial expression; usually mild and self-limited	Reassurance; observation in mild cases. May be corrected by injecting some extra toxin into the active area of the muscle	[16]
Perioral dysfunction: lip ptosis, drooling	Described as rare	Impaired speech, drooling, difficulty drinking and eating, particularly relevant in neurological patients	Conservative management; explanation and reassurance	[16]
Local injection-site reactions: pain, erythema, oedema, bruising	Pharmacovigilance study report pain 9.3%, swelling 6.4%, erythema 1.8%, bruising 1.2%	Generally mild and transient; can affect the acceptability of repeated treatment cycles	Ice packs, gentle compression, simple analgesics	[32,18,29,27,22,17]
Headache	Pharmacovigilance study reports 4.3%	Usually transient; occasionally prolonged, requiring analgesics and clinical follow-up	Symptomatic treatment with standard analgesics (NSAIDs or opioids). Most are mild and spontaneously resolve within a few days	[32,4,27,22,17]
Other events: fatigue, flu-like symptoms, weakness, dyspnoea, dysphagia, dizziness, allergy	Pharmacovigilance study reports fatigue 2.8%, flu-like illness 1.4%, dyspnoea 1.3%, dysphagia 1.2%, allergy/urticaria 3.4%	Predominantly mild; may require hospital care	Supportive care; consider hospital admission if severe	[25,17,22]

Discussion

Across both aesthetic and therapeutic settings, the literature consistently shows that BoNT is effective and generally well tolerated, with most adverse events being mild and transient [10,12]. Although most adverse events are transient and occur at low frequency, their functional and aesthetic consequences may be disproportionate, particularly when involving vision, facial expression, or appearance. Even primarily cosmetic changes, such as facial asymmetry or eyebrow deformities, have been associated with patient distress and reduced quality of life following facial injections [10,21].

Periocular involvement, such as upper eyelid ptosis, even when limited to a few millimetres, can restrict the superior visual field and interfere with reading and other near-vision tasks, depending on baseline visual reserve and individual tolerance [19,25]. Diplopia, although less frequent, is often more disabling, compromising walking, driving, and any activity that relies on stable binocular vision [20,30]. Dry eye and ocular surface changes have been reported only in isolated cases, possibly under-recognized, but are associated with pain, foreign-body sensation, and risk of keratopathy if not promptly recognized [19,31].

Management strategies for periocular complications remain largely conservative, but upper eyelid ptosis is the condition for which the most detailed approaches are described. Topical alpha-adrenergic eye drops, particularly apraclonidine 0.5%, one or two drops, two to four times daily, are recommended as first-line treatment, based mainly on expert consensus and small observational studies. They can provide 1-2 mm of compensatory lid elevation with functional improvement within a few days [18,27,34]. More recently, oxymetazoline 0.1% was approved by the FDA in 2020 as the first pharmacological treatment for acquired blepharoptosis in adults. Randomized phase III trials in adults with acquired blepharoptosis, not specifically limited to BoNT-induced cases, demonstrated that once-daily oxymetazoline produces rapid eyelid elevation within 5-15 minutes, with mean marginal reflex distance (MRD) gains of approximately 0.6-1.1 mm and associated improvements in superior visual field, sustained for 2-6 hours and maintained over six weeks of continuous use [35,36]. Alternative agents such as phenylephrine, naphazoline, or brimonidine are suggested in some reports, chosen according to ocular comorbidities and availability [16,18,29].

In addition to topical pharmacological therapy, several authors have proposed pretarsal BoNT injection as a targeted strategy to manage eyelid ptosis, an approach that requires advanced expertise and carries the risk of misapplication if performed outside specialist settings. In a small case series of involutional mild ptosis, transcutaneous injection of 2-3 units of BoNT placed approximately 2 mm above the lash line at medial and lateral sites produced MRD improvements of 0.8-1 mm with satisfactory outcomes and no major adverse effects [37]. More recently, a series of eight patients with severe botulinum-induced blepharoptosis reported that pretarsal injections of 2-4 units of onabotulinumtoxinA across two to three superficial points along the medial and lateral limbus, used alone or in combination with oxymetazoline 0.1%, achieved a mean eyelid elevation of 3.4 mm and full resolution of ptosis within 10-14 days without complications [38]. Although these findings are encouraging, the evidence is limited to small case series without control groups and with non-standardized outcome assessment, highlighting the need for careful patient selection and well-designed controlled studies.

More pronounced cases have occasionally been managed with adjunctive measures such as lymphatic drainage, low-voltage electrical stimulation, or radiofrequency; however, these approaches are supported only by anecdotal reports, lack controlled evidence, and cannot currently be regarded as evidence-based management options [19,26].

Ocular lubrication and surface protection are used for dry eye, whereas diplopia is usually managed with temporary occlusion or prisms and observation until spontaneous recovery [19,20,31]. In selected cases, particularly when binocular disturbance is functionally significant, chemodenervation of the ipsilateral antagonist muscle with BoNT-A may be considered, as described in isolated reports; in the case reported by Isaac et al., 2.5 U of onabotulinumtoxinA were injected into the ipsilateral medial rectus under topical anaesthesia, resulting in marked improvement within 2-7 days. Given the risk of inducing further ocular misalignment, this intervention should be reserved for experienced clinicians and strictly individualized [30].

Perioral complications, while rare, are functionally meaningful. Misplacement or excessive dosing near the orbicularis oris can cause oral incompetence, drooling, and speech disturbance, which may disproportionately affect patients with pre-existing neurological impairment. Because published studies describe only conservative management and expectant recovery, and no pharmacological or interventional alternatives have been systematically evaluated, the lack of structured treatment algorithms represents a relevant evidence gap [11,18].

Local reactions and headache remain the most frequent adverse events and are generally trivial, with a well-defined symptomatic approach [17,27,32]. Systemic adverse events, although uncommon, are consistently reported in pharmacovigilance data and include fatigue, flu-like symptoms, generalized weakness, dyspnoea, and dysphagia, highlighting that facial BoNT cannot be regarded as a purely local intervention. However, detailed management guidance is scarce, and published recommendations are limited to supportive care and timely hospital evaluation in severe presentations [17,33]. This lack of structured protocols represents another important limitation in the existing literature.

Taken together, these findings indicate that most complications are preventable through sound knowledge of facial anatomy, conservative dosing, and appropriate patient selection [10,12,39]. However, the evidence base is dominated by narrative reviews, single-centre case series, and spontaneous report databases, with very few prospective or comparative studies. Future research should prioritize the standardized reporting of complications and clearer management pathways, particularly for periocular complications, perioral dysfunction, and systemic adverse events.

## Conclusions

Facial BoNT injections are generally safe, yet periocular, perioral, and systemic complications can occur and may have a disproportionate functional impact in patients treated for neurological conditions. Management described in the literature is largely symptom-oriented and conservative, which may reflect the limited availability of validated corrective interventions rather than evidence that conservative care is superior. The available literature supports a predominantly conservative, symptom-oriented approach: alpha-adrenergic eye drops, particularly apraclonidine 0.5%, remain the most commonly reported first-line option for toxin-induced ptosis, while oxymetazoline 0.1%, FDA-approved for the treatment of acquired blepharoptosis, offers an additional pharmacological alternative with demonstrated short-term improvements in eyelid position and visual field. Management of other complications similarly relies on symptomatic measures, including ocular lubrication for dry eye, occlusion or prisms with observation for diplopia, reassurance and expectant recovery for perioral weakness, and routine analgesia for local injection-site reactions and headache. However, most recommendations are founded on narrative reviews, case reports, or pharmacovigilance data rather than controlled studies. Future work should therefore prioritize prospective registries and comparative clinical studies to better define complication incidence, strengthen management algorithms, and optimize prevention and treatment strategies for facial BoNT complications in clinical practice.
